# Analysis of Blood Biochemistry of Free Ranging and Human-Managed Southern White Rhinoceros (*Ceratotherium simum simum*) Using the i-STAT Alinity v®

**DOI:** 10.1155/2021/2665956

**Published:** 2021-07-19

**Authors:** Shweta Trivedi, Christina M. Burnham, Christian M. Capobianco, Christiaan Boshoff, Yaxin Zheng, Jordan Wood Pettiglio, Kimberly Ange-van Heugten, Heidi D. Bissell, Larry J. Minter

**Affiliations:** ^1^Department of Animal Science, North Carolina State University, 120 W Broughton Drive, Raleigh, NC 27607, USA; ^2^College of Veterinary Medicine, North Carolina State University, 1060 William Moore Drive, Raleigh, NC 27606, USA; ^3^SA World Vets, Gravelotte, South Africa; ^4^Department of Statistics, North Carolina State University, 2311 Stinson Drive, Raleigh, NC 27607, USA; ^5^Busch Gardens Tampa Bay, 10165 McKinley Drive, Tampa, FL, USA; ^6^Hanes Veterinary Medical Center, North Carolina Zoo, 4401 Zoo Parkway, Asheboro, NC 27205, USA

## Abstract

Handheld point-of-care blood analyzers deliver rapid results for biochemical and hematologic parameters, making them very useful in veterinary clinics and in fieldwork applications. This study compared the biochemical and hematologic parameters generated by the novel point-of-care analyzer i-STAT® Alinity V CHEM8+ cartridge between human-managed and free ranging populations of southern white rhinoceros (*Ceratotherium simum simum*). In addition, a novel reference interval for ionized calcium (iCa), a parameter of diagnostic and prognostic importance, was established for southern white rhinoceros. Blood samples were obtained from 10 managed (6 at NC Zoo and 4 at Busch Gardens Tampa Bay in 2019) and 30 free ranging white rhinoceros (collected in South Africa between 2018 and 2019) and analyzed using the i-STAT. Multiple parameters were higher (*P* < 0.05) in free ranging versus managed animals including potassium, blood urea nitrogen, creatinine, glucose, hematocrit, and hemoglobin. Conversely, iCa concentrations were higher (*P* < 0.05) in the managed populations of white rhinoceros. The RI determined for iCa was 1.36–1.56 mmol/L, with a mean of 1.46 mmol/L, and was determined using the guidelines from the American Society for Veterinary Clinical Pathology. There was no difference in anion gap, chlorine, total carbon dioxide, or sodium between the populations. Seasonality and locality of sampling as well as diet may be contributing factors to the higher iCa concentrations in managed rhinoceros. The six elevated parameters in free ranging rhinoceros are likely attributable to dehydration compounded by capture stress. This data provides insight into the state of several biochemical and hematologic parameters in southern white rhinoceros and will allow veterinarians to better assess the health of both managed and free ranging populations.

## 1. Introduction

The southern white rhinoceros (*Ceratotherium simum simum*) is a paragon of successful wildlife management and conservation; after years of intense poaching pressure left only 20 animals in the wild in 1885, conservation efforts led to a resurgent population last estimated at over 20,000 individuals [1, 2]. Despite this, southern white rhinoceros are listed as Near Threatened by the IUCN due to the continually high black market demand for rhino horn and the subsequent increase in poaching levels [3]. Due to the uncertain future of southern white rhinoceros in the wild, it is critical that a healthy population be maintained in human care to ensure their long-term survival. Recent advancements in veterinary technology help facilitate these ex situ conservation efforts.

The newest tools in the arsenal of veterinary research are portable point-of-care blood analyzers such as the i-STAT® Alinity V system (Zoetis, New Jersey 07932, USA), which uses test cartridges to analyze a range of physiologic measurements for comparison against species-specific reference intervals (RIs). Previous studies have validated the use of point-of-care analyzers with white rhinoceros in the field for most measures [4–7]. Portable analyzers are of particular use in veterinary fieldwork as they are capable of quickly determining the likely outcome of an injured animal depending on generated prognostic parameters and are helpful in the monitoring of the health of populations of wild animals. Assisting in triage of free-ranging animals injured in poaching or human-animal conflicts and indicating whether that animal is able to survive further intervention allow conservators to allocate resources more effectively.

Despite their practicality, both point-of-care and laboratory analyzers generate analyzer-specific values, and as such most values are not comparable across different platforms [7–9]. Thus, it is important for there to be comprehensive publication of reference intervals (RIs) from all point-of-care analyzers used in veterinary medicine. Framework developed by the American Society for Veterinary Clinical Pathology (ASVCP) allows for standardization of species-specific RIs [10]. Biochemical and hematologic parameters for southern white rhinoceros generated by the CHEM8+ (Zoetis, New Jersey 07932, USA) test cartridge using the i-STAT Alinity V have yet to be compared between free ranging and human-managed populations. Similarly, there are no published RIs for ionized calcium (iCa) in southern white rhinoceros using any analyzer [11].

The objectives of this research are twofold: (1) to compare the nine biochemical and two hematologic parameters generated using the i-STAT Alinity V CHEM8+ cartridge between free ranging and managed populations of southern white rhinoceros and (2) to establish reference intervals for previously undescribed iCa concentrations in the blood of southern white rhinoceros using an i-STAT Alinity V handheld blood analyzer. We hypothesized that iCa concentrations would be higher in human-managed animals when compared to free-ranging conspecifics due to diet and seasonality of sampling.

## 2. Materials and Methods

### 2.1. Sample Collection

Whole blood samples were collected from a free ranging population of southern white rhinoceros in South Africa and from managed populations at two North American zoos. Samples from 30 male free ranging rhinoceros were obtained during anesthesia procedures to conduct routine herd health and management in May and June of 2018 (*n* = 16) and June of 2019 (*n* = 14). The sample population consisted of 18 adult and 12 subadult animals. Rhinoceros are categorized as subadults at 3.5–7 years of age, with females categorized as adults at 7+ years and males at 8+ years of age [12]. Rhinoceros were darted in the greater Kruger region of South Africa from helicopter. Each animal was anesthetized with a combination of 2.0–3.5 mg of etorphine (M99, Voluplex, Centurion 0157, South Africa) and 20–40 mg of azaperone (Stresnil, Elanco, Kempton Park 1619, South Africa) for subadults and 3.5–4.2 mg of etorphine and 40 mg of azaperone for adults [13]. All animals appeared to be healthy based upon body condition and limited physical examination. It took an average of 4 to 8 minutes for animals to become recumbent after darting, during which time they were walking or running freely for 100 to 150 meters. Animals were placed in lateral recumbency during anesthesia and no supplemental oxygen was provided throughout the procedure. Cardiopulmonary values were monitored while the animals were under anesthesia, including heart rate (HR) and respiration rate (RR). A complete blood cell count (CBC) was also performed. Blood samples were obtained within five minutes of immobilization via the posterior auricular vein and were placed in green top (lithium heparinized) BD Vacutainer® tubes (Becton, Dickenson, and Company, Franklin Lakes, NJ 07417, USA).

Whole blood samples were obtained from the radial veins of 10 managed southern white rhinoceros (1 male, 9 females) during routine nonimmobilized venipuncture training. Samples were collected from 6 animals at the North Carolina Zoo (1 male, 5 females) and 4 female animals from Busch Gardens Tampa Bay in February of 2019 and April of 2019, respectively. All animals were deemed to be healthy by their respective animal health teams at the time of sample collection. The diet of North Carolina Zoo southern white rhinoceros includes one bale of timothy hay per animal daily and three pounds of Mazuri Wild Herbivore High Fiber diet (PMI Nutrition International, St. Louis, Missouri 63108, USA) per animal supplied daily. Timothy hay cubes and pellets, orchard grass hay, and alfalfa hay are supplemented during training and enrichment. The Busch Gardens Tampa Bay rhinoceros are provided 3/4ths of a bale of timothy hay per animal daily. Both populations have access to fresh pasture for grazing while on exhibit.

The study protocol was approved by the North Carolina Zoo Research Committee, the Busch Gardens Research Committee, and the Institutional Animal Care and Use Committee at North Carolina State University College of Veterinary Medicine (Protocol #19-543-O).

### 2.2. Sample Analysis

Samples were kept in a cooler on ice blocks to ensure sample viability until processing could occur. Samples were evaluated for hemolysis before processing, and no samples with significant hemolysis were used. Blood chemistry profiles were performed using i-STAT CHEM8+ cartridges in the i-STAT Alinity V within ten minutes of collection from managed animals and within two hours of collection for free ranging animals. Parameters tested by the CHEM8+ cartridge include sodium (Na), potassium (K), chlorine (Cl), total CO_2_ (TCO_2_), blood urea nitrogen (BUN), creatinine, glucose, ionized calcium (iCa), anion gap (AnGap), hematocrit (Hct), and hemoglobin (Hgb). For each sample, 0.2 mL of whole blood was loaded onto the cartridge. Quality control of the i-STAT Alinity V was performed as per the manufacturer's guidelines [14].

### 2.3. Statistical and Comparative Analysis

A comparison of adult and subadult biochemical and hematologic values yielded no significant difference between the two age groups; as such, adult and subadult values from free ranging and managed populations were combined for comparative analysis.

The establishment of the RI for iCa was completed by using the nonparametric bootstrapping method recommended for sample sizes of 40 or more by guidelines from the American Society for Veterinary Clinical Pathology after examination of the data found that it did not follow a normal distribution [10]. Horn's algorithm using Tukey's interquartile fences was used to identify the outliers. One outlier value (1.19) was identified for iCa. After examining the data for normality using the Shapiro–Wilk test, the data distribution for iCa was found to not follow normal distribution. Log and Box-Cox transformation options were tested for the variable [10]. Ionized calcium values were still skewed after transformation; therefore, a parametric method could not be applied to establish the RI for iCa. Bootstrapping methods were thus used to generate the 90% RI.

The nine biochemical and two hematologic parameters from the two populations were examined for normality using the Shapiro–Wilk test. Only Na, K, and Cl were normally distributed [10]. Therefore, two sets of *t*-tests were applied on Na, K, and Cl to identify the mean differences. The other variables (TCO_2_, BUN, creatinine, glucose, iCa, AnGap, Hct, and Hgb) were analyzed using Wilcoxon Signed Rank Test. All the *P*-values in the statistical analyses were adjusted using Bonferroni correction. All analyses were performed in R (R Core Team, 2019) and RStudio (version 1.0.143).

## 3. Results

Biochemical and hematologic values concentrations were measured in a total of 40 free ranging (*n* = 30) and managed (*n* = 10) southern white rhinoceros of varying sexes and ages (Table 1). Multiple parameters (mean ± standard error of the mean (SEM)) were higher (*P* < 0.05) in free ranging versus managed animals including K (5.70 ± 0.15), BUN (15.77 ± 0.51), creatinine (1.53 ± 0.03), glucose (125.03 ± 5.61), Hct (44.44 ± 1.30), and Hgb (15.11 ± 0.44) (Table 2). There was no statistically significant difference in AnGap, Cl, TCO_2_, or Na between managed and free ranging populations.

The RI for iCa was determined to be 1.36–1.56 mmol/L, with a mean of 1.46 mmol/L. Ionized calcium concentrations were significantly higher in the managed population of southern white rhinoceros compared to free ranging conspecifics (*P* < 0.05) (Table 2).

## 4. Discussion

Six parameters were found to be significantly higher (*P* < 0.05) in free ranging versus managed animals, including K, BUN, creatinine, glucose, Hct, and Hgb (Table 2). Differences in five of these parameters (K, BUN, creatinine, Hct, and Hgb) are likely attributable to dehydration due to lack of accessible water, as animals were sampled during the dry season in the Greater Kruger region of South Africa. This effect was likely compounded by capture stress, as the free ranging rhinoceros had to be anesthetized for routine health procedures and thus were pursued and darted from a helicopter.

Higher K values in free ranging rhinoceros contrasts with previously published reference values for K, the mean of which was higher in managed animals (4.8 mmol/L) than in free ranging animals (4.2 mmol/L) [11]. There are no published reference intervals for BUN nor creatinine for managed southern white rhinoceros using any analyzer. A previous study had established the mean BUN concentration for free ranging southern white rhinoceros (*n* = 181) in Kruger National Park as 11.4 mg/dL using the VetScan2 (Zoetis, New Jersey 07932, USA) [13]. Creatinine reference values for free ranging southern white rhinoceros in Kruger National Park have been established by using two different point-of-care analyzers: the VetTest (IDEXX Laboratories, Inc., Westbrook, ME, USA) and the Cobas Integra 400 Plus (Roche Products [Pty] Ltd., Basel, Switzerland); the mean values were 1.71 mg/dL and 1.57 mg/dL, respectively (*n* = 51) [7]. Despite similarities in values between the Cobas and i-STAT analyzers, the two devices use different methods for analyzing creatinine concentrations; the Cobas utilizes the Jaffé method with alkaline picrate, while the i-STAT utilizes creatine amidinohydrolase [7, 15]. While the VetTest also uses a creatine amidinohydrolase assay, the creatinine value generated was less similar than that of the i-STAT. There is no published reference interval for creatinine from managed southern white rhinoceros.

Both mean Hct and Hgb concentrations were higher in free ranging southern white rhinoceros when compared to managed rhinoceros (Table 2). While elevated Hct and Hgb values are likely due to dehydration, they may also coincide with polycythemia [16]. High Hgb concentrations can also be a result of hypoxia and while the anesthetized rhinoceros were not provided with supplemental oxygen, it is unlikely that there was sufficient time for Hgb concentrations to increase due to lack of oxygen during immobilization. Our free ranging Hct value is very similar to a previously published value of 44.46% for free ranging white rhinoceros in Kruger National Park (*n* = 115) [17]. The previously published mean value for Hgb in free ranging southern white rhinoceros is 14.62 g/dL [17]. There are no published reference values for Hct nor Hgb from managed southern white rhinoceros.

Mean glucose concentrations were higher in free ranging southern white rhinoceros when compared to managed rhinoceros (Table 2). The free ranging rhinoceros in this study were followed via helicopter before being darted; thus it follows that glucose would be released from storage in the liver into the bloodstream, stimulated by epinephrine produced during an acute stress response [18]. In contrast, the managed animals were sampled at rest without chemical restraint. A study by Hooijberg et al. [7] published comparable blood glucose reference values for free ranging white rhinoceros (*n* = 51) using the VetTest (138.6 mg/dL) and Cobas (128.2 mg/dL) analyzers. The animals in that study were similarly darted from a helicopter, and a secondary population of injured rhinoceros were also sampled; this population was likely also experiencing some degree of stress [7]. There is no published reference interval for glucose from managed southern white rhinoceros.

The AnGap parameter was of particular interest, as it can be used in the field as a prognostic indicator in cases involving wounds and poaching-inflicted fracture repair using external fixators. Comparative analysis of AnGap concentrations in managed versus free ranging populations of southern white rhinoceros revealed no significant differences. This is unsurprising and corroborates with current literature, which shows that a high AnGap in an individual is indicative of some form of metabolic acidosis and is associated with an increased all-cause mortality risk [19, 20]. All animals in the sampling population were healthy, and so they were not in a state of metabolic acidosis. Regardless, lactate remains the most relevant indicator of increased mortality risk in veterinary practice [21]. In addition to AnGap, there were no significant differences in concentrations of Na, Cl, and TCO_2_ between the populations.

The RI calculated in this study is specific to blood chemistry results from the i-STAT Alinity V handheld analyzer. There are currently no published RIs for iCa in any rhinoceros species to serve as a comparison to the determined interval. Horses are generally accepted as the domestic model for white rhinoceros and do have a published RI for iCa using the i-STAT Alinity v (1.25–1.75 mmol/L) which is much broader than the interval generated from rhinoceros in this study [14]. This is likely due to the RI for horses having been generated from thousands of individuals, while the RI established here is limited in scope.

Statistical analysis showed that iCa concentrations were significantly higher in managed southern white rhinos when compared to free ranging populations. Ionized calcium, also referred to as “free calcium,” is the measure of free hydrated calcium cations in the blood; this contrasts with total calcium concentration, which indicates the total amount of calcium in the blood, including both iCa and calcium bound to proteins [22, 23]. Ionized calcium represents the physiologically active portion of the total calcium value and is thus the more relevant blood calcium reading. While statistically significant, it is unlikely that the difference in iCal levels between free ranging and managed rhinoceros is clinically significant. This difference may be due to seasonality and locality of sampling, as iCa is linked to vitamin D, which has been shown to fluctuate seasonally in eastern black rhinoceros (*Diceros bicornis michaeli*) [24]. Free ranging southern white rhinoceros in this study were sampled during the winter month of June in the Southern Hemisphere, which may account for the lower iCa concentrations in comparison with samples from managed rhinoceros collected during the spring in the northern hemisphere. It was observed in the greater one-horned rhinoceros (*Rhinoceros unicornis*) that circulating concentrations of calcifediol were statistically higher during the spring and summer than in the winter [25]. This effect may have been compounded by the animals being housed inside during the adverse winter weather [25].

Diet may have also played a role in the difference in iCa concentrations between free ranging and managed animals. Free ranging animals are subject to intra- and interspecies competition for grazing areas, which is exacerbated by drought, fire, and anthropic effects [26, 27]. As such, nutritious grasses critical to grazing megafauna may not have been readily available to the free ranging study population around the time of sampling, impacting nutrition and thus blood mineral values. In contrast, the diets of managed white rhinoceros are both consistent and nutrient-rich. Past studies have shown that grasses and forage in tropical regions are of lower quality than those of temperate regions; similarly, grasses in South Africa are chronically deficient in minerals, including calcium [28–30]. A comparison of the nutritional quality of South African grasses preferentially grazed by free ranging southern white rhinoceros to that of grasses provided in a European zoo provided further validity, as calcium and phosphorus concentrations in the South African grasses were half that of the grasses at the European zoo [29]. Grasses consumed by rhinoceros at a temperate North American zoo are likely higher in calcium content than those consumed by free ranging conspecifics in South Africa, which may account for the higher iCa concentrations detected. In addition, the population of managed rhinoceros at the NC Zoo are offered a nutritionally complete pellet as part of their diet which would mitigate any deficiencies.

Limitations of this study include a small sample size and skewed sex ratios; all free ranging animals were male, versus a majority female managed population. The effect of age or sex on iCa concentrations in southern white rhinoceros thus remains unknown without further study and a larger sample size. Another limitation to consider is that all free ranging animals had to be anesthetized without respiratory support for sample collection and were thus subjected to a significant number of other physiological derangements inherent in anesthesia. These stressors, however, would likely not affect iCa concentrations in the time span between recumbency and recovery. Lastly, free ranging and managed animals were located in different geographical regions, with free ranging rhinoceros having unmitigated access to sunlight and grazing year-round while one of the managed groups of rhinoceros was housed inside during adverse winter weather and low temperatures.

## 5. Conclusions

The study provides insight into the state of multiple biochemical and hematologic parameters in different populations of southern white rhinoceros and will allow veterinarians to better assess the health of both managed and free ranging populations using point-of-care analyzers in field. The findings of this study also show that iCa levels are higher in managed southern white rhinoceros when compared to free ranging conspecifics, likely due to sampling seasonality and diet. Additional research is necessary to understand the exact effects of these variables on iCa concentrations in the blood of southern white rhinoceros.

## Figures and Tables

**Table 1:** Summary of sex and age distributions of free ranging and managed southern white rhinoceros (*Ceratotherium simum simum*) analyzed via the biochemical handheld analyzer.

Summary	Distributions	Free ranging (*n* = 30)	Managed (*n* = 10)
Sex (*n*)	Male	30	1
	Female	0	9

Age	Adult	18	10
	Subadult	12	0

**Table 2:** Descriptive statistics and ionized calcium reference interval for biochemical values in free ranging and managed southern white rhinoceros (*Ceratotherium simum simum*) analyzed via the handheld analyzer.

Biochemistry	RI	Free ranging							Managed						
		*n*	Mean	±	SEM	Median	Min	Max	*n*	Mean	±	SEM	Median	Min	Max
Na (mmol/L)	—	30	131.1	±	0.58	131	125	137	10	132.5	±	0.67	133	128	135
K (mmol/L)	—	30	5.70	±	0.15^b^	5.35	4.5	7.3	10	4.24	±	0.08^c^	4.3	3.8	4.6
Cl (mmol/L)	—	30	95.37	±	0.64	96	90	102	10	92.60	±	0.88	93	88	96
TCO_2_ (mmol/L)	—	30	27.17	±	0.99	28.5	11	34	10	30.30	±	0.87	30	26	34
BUN (mg/dL)	—	30	15.8	±	0.51^b^	16	10	20	10	8.1	±	1.03^c^	6.5	5	13
Creatinine (mg/dL)	—	30	1.53	±	0.03^b^	1.55	1.3	1.9	10	1.29	±	0.08^c^	1.35	0.8	1.7
Glucose (mg/dL)	—	30	125.0	±	5.61^b^	121	82	194	10	89.5	±	11.05^c^	74.5	70	181
AnGap (mmol/L)	—	30	15.3	±	0.78	15	10	26	10	14.8	±	0.66	15.5	12	17
Hct (%)	—	27	44.44	±	1.30^b^	44	37	71	10	34.60	±	1.96^c^	33	29	48
Hgb (g/dL)	—	27	15.11	±	0.44^b^	15	12.6	24.1	10	11.75	±	0.66^c^	11.2	9.9	16.3
iCa (mmol/L)	1.36–1.56	30	1.43	±	0.02^b^	1.46	1.19	1.57	10	1.52	±	0.01^c^	1.52	1.46	1.57

Values in the same row with different superscripts are significantly different (*P* < 0.05). RI: reference interval; SEM: standard error of the mean.

## Data Availability

The datasets used to support the findings of this study are available from the corresponding author upon request.
